# Dirac plasmon-assisted asymmetric hot carrier generation for room-temperature infrared detection

**DOI:** 10.1038/s41467-019-11458-5

**Published:** 2019-08-02

**Authors:** Alireza Safaei, Sayan Chandra, Muhammad Waqas Shabbir, Michael N. Leuenberger, Debashis Chanda

**Affiliations:** 1 Department of Physics, University of Central Florida, Orlando, FL 32816 USA; 2 NanoScience Technology Center, University of Central Florida, Orlando, FL 32826 USA; 3 CREOL, The College of Optics and Photonics, University of Central Florida, Orlando, FL 32816 USA

**Keywords:** Optical properties and devices, Optical properties and devices, Optical properties and devices

## Abstract

Due to the low photon energy, detection of infrared photons is challenging at room temperature. Thermoelectric effect offers an alternative mechanism bypassing material bandgap restriction. In this article, we demonstrate an asymmetric plasmon-induced hot-carrier Seebeck photodetection scheme at room temperature that exhibits a remarkable responsivity of 2900 VW^−1^, detectivity of 1.1 × 10^9^ Jones along with a fast response of ~100 ns in the technologically relevant 8–12 µm band. This is achieved by engineering the asymmetric electronic environment of the generated hot carriers on chemical vapor deposition grown large area nanopatterned monolayer graphene, which leads to a temperature gradient of 4.7 K across the device terminals for an incident power of 155 nW, thereby enhancing the photo-thermoelectric voltage by manifold compared to previous reports. The results presented outline a strategy for uncooled, tunable, and multispectral infrared detection.

## Abstract (web summary)

Designing electronically asymmetric environment across monolayer graphene enhances the photo-thermoelectric effect and enables efficient infrared photodetection. Here, the authors report a photodetector based on CVD grown nano-patterned graphene with high responsivity of ~2900 V/W within 8–12 µm wavelength regime by Dirac plasmon-assisted hot carrier generation at room-temperature.

## Introduction

Two-dimensional (2D) materials, especially graphene has shown a lot of potential as a candidate material for infrared detection. An ultrafast (~ps) infrared detection process is to excite hot-carriers in absence of carrier-phonon scattering^1–3^ and probe the electronic temperature of graphene for infrared sensing by exploiting the photo-thermoelectric effect^4–6^. It is known that upon illumination, the intrinsic carrier temperature of graphene increases by means of hot carrier generation that manifests as a Seebeck voltage (Δ*V*)^1,3,4,7^. Despite the proof-of-concept demonstrations, so far, due to the modest Seebeck coefficient of graphene ~100 µVK^−1^, it has not been possible to harness this effect as an effective approach that can rival contemporary technologies^1,2,4^.

**Fig. 1:**
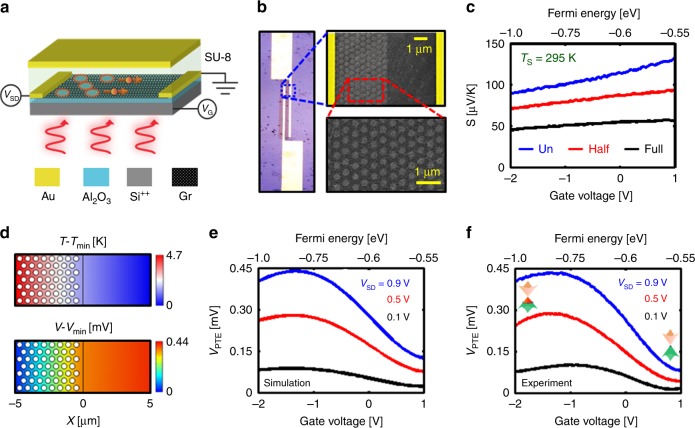
Design and performance. **a** Schematic of the device architecture of the plasmon-assisted hot carrier generation on an asymmetrically nanopatterned graphene. Arrows on the graphene sheet show hot carrier diffusion processes. **b** The optical (left) and scanning electron microscope (right) images of the half-patterned graphene sample. **c** Seebeck coefficient (*S*) of the unpatterned, half-patterned and full-patterned graphene samples (*P* = 600 nm and *D* = 400 nm) as a function of gate voltage at room temperature. **d** The simulated temperature (top) and potential (bottom) profiles of the graphene detector at *E*_F_ = −0.85 eV, λ_res_ = 8.15 μm and *V*_SD_ = 0.9 V. The simulated, **e** and measured, **f** D.C photo-thermoelectric voltage (*V*_PTE_) of the graphene detector as a function of Fermi energy for the different bias voltages. Blue, red and black diagrams correspond to the source-drain voltages of 0.9 V, 0.5 V, and 0.1 V, respectively. (Un: unpatterned—Half: half-patterned—Full: full-patterned)

The motivation of the present work is to identify avenues on how the temperature gradient (Δ*T*) of the charge carriers can be engineered with minimal effect on the lattice temperature in order to enhance Seebeck voltage generation for highly sensitive, spectrally tunable, fast infrared detection in the LWIR band at room temperature. The carrier temperature of graphene at a specific spectral range can be manipulated by the plasmonic excitation of Dirac fermions which can be controlled by electrostatic tuning of the Fermi level. In our recent work, spectrally tunable infrared absorption of ~60% in the LWIR was demonstrated for nanopatterned monolayer graphene coupled to an optical cavity^8^. At resonance, due to the strong confinement of electric field at the discrete nanoresonator edges, the electronic system of graphene heats up by means of boundary-assisted intraband Landau damping to generate hot-carriers^6,9^. Although the hot-carrier generation develops a change in conductance of graphene, the resultant photoresponse arising from Δ*T* is limited by the theoretical Seebeck coefficient of graphene. The key to an efficient photo-thermoelectric device is the generation of high temperature gradient which we demonstrate in our asymmetric plasmon-induced hot-carrier Seebeck photodetector. In the asymmetric device (Fig. 1a) the active detector area consists of a partially nanopatterned monolayer graphene, such that there exists a temperature gradient between the hot carriers in the patterned and the unpatterned regions. In addition to the temperature rise from intrinsic intraband hot carrier generation in graphene, the plasmon-assisted hot carriers in the patterned section further enhances the effective temperature gradient across the source-drain contacts. Owing to this enhanced record Δ*T* generation, the fabricated LWIR detectors exhibit an outstanding room temperature responsivity of 2900 VW^−1^, detectivity (D*) of 1.1 × 10^9^ Jones along with a fast response of ~100 ns.

The two important parameters that determine a detector’s performance are (a) detectivity and (b) response time. The uncooled detector devices or bolometers working at room temperature are very slow with the response time of *τ* ~ ms^10,11^, while having the detectivity of D* ~ 10^8^ to 10^9^ Jones. Furthermore, photoconductive or photovoltaic detectors have limited operation bandwidth determined by their bandgap. Keeping the above-mentioned attributes in perspective, the proposed frequency-tunable graphene detector in this work not only offers spectroscopic detection with much lower response time (~ns), but also maintains a comparable high detectivity of ~10^9^ Jones.

## Results

### Plasmon assisted hot carrier generation

The plasmon assisted photo-thermoelectric (PTE) detector design and the fundamental electronic processes involved are schematically shown in Fig. 1a. A monolayer graphene between the source and drain electrodes forms the active detector surface where a section of the graphene is patterned into a hexagonal array of holes (Supplementary Fig. 1). In doing so, an asymmetry is inherently introduced into the system that assists infrared detection as explained below. Selective patterning of graphene allows enhanced infrared absorption^8,12^ arising from localized Dirac plasmon excitations associated with intraband transitions. As a result, a gradient is introduced into the charge carrier density of the graphene channel across the source-drain electrodes. This creates a temperature difference across the graphene channel that as we discuss later in details plays the key role in infrared detection mechanism. Based on the applied source-drain voltage (*V*_SD_), henceforth called bias voltage, and the gate voltage (*V*_G_), multiple electronic processes work in tandem or against each other that influence the detector response. The graphene channel width is chosen to be 10 µm which is comparable to the diffusion length of the charge carriers^3^. However, in order to enhance carrier collection, the graphene channel width is elongated to maintain an effective active area of 2000 µm^2^ as shown in Fig. 1b-left. The optimized nanopattern geometry which is separately reported in our recent publication^8^, of period *P* = 600 nm and hole diameter *D* = 400 nm was chosen in the detector fabrication (Supplementary Fig. 6). The scanning electron microscope (SEM) images (Fig. 1b-right) reveal the hexagonal array of holes in the patterned section along the graphene channel.

The Fermi energy of graphene at 0 V is determined (Supplementary Fig. 2) to be −0.6 eV which suggests that the graphene sheet is self-doped to be p-type. Such self-doping effects have been reported earlier^13,14^ that arises due to the residual impurities on the graphene surface. In addition, the Al_2_O_3_ gate dielectric is known to enhance p-type doping in graphene^15,16^ (Supplementary Figs. 8 and 19). Therefore, as the gate voltage is swept from +1 V to −2 V, the hole concentration on graphene increases consistent with a change in Fermi energy from −0.55 eV to −1.0 eV. Our experiments show that nanopatterning decreases the carrier mobility of graphene. Consequently, it is imperative to study how the thermoelectric properties of graphene are modified due to nanopatterning. This will allow us to gain insight into the fundamental working mechanism of the detector.

The experimentally extracted Seebeck coefficient (*S*) of different graphene devices as a function of Fermi energy is shown in Fig. 1c which proves the nanopatterning decreases the Seebeck coefficient (Supplementary Figs. 3 and 4). It can be observed that by electrostatically increasing the p-doping, as the Fermi energy is lowered, the Seebeck coefficient decreases which is consistent with previous reports^17,18^ on graphene doped away from the charge neutrality point. It can also be noticed that with decrease in temperature, *S* diminishes (Supplementary Figs. 4 and 5) which makes the present detection scheme more efficient at room temperature.

### Photovoltage generation

Upon illumination with infrared light the electronic properties across the half-patterned graphene channel exhibit contrasting electronic behavior. When light is incident on the unpatterned section of the graphene channel coupled to an optical cavity, the light absorption is a modest ~3%, however in the patterned section, owing to Dirac LSP excitations, ~60% light is absorbed by way of strong confinement of electric field near the nanohole edges (Supplementary Fig. 7)^8^. Supplementary Figure 7 demonstrates that the absorption peak location was tunable over ~2.5 µm in the 8–12 µm band by electrostatic doping. The normal-angle reflection spectra (*R*) of the fabricated cavity-coupled graphene nanohole array absorber were measured using a Microscope-coupled Fourier transform infrared spectrometer (FTIR) (Hyperion 1000—Vertex 80, Bruker Inc.). The light reflection from the absorber stack without patterned graphene, i.e. Si^++^ (100 μm)/Al_2_O_3_ (15 nm)/SU-8 (1.3 μm)/Al_2_O_3_ (50 nm)/gold (200 nm) was taken as the reference and the light absorption spectra was calculated as *A* = 1-*R*.

Once excited, the LSPs dissipate energy through various damping pathways like phonon emission^9,19^, bulk scattering or carrier-carrier edge scattering^6,9,19^ that influence either the lattice or carrier temperature of graphene depending on which of the above-mentioned damping mechanisms are dominant. Since the LSP excitations (115–155 meV) in our case occur at energies lower than the optical phonon energy of graphene (200 meV)^20^ and far from that of the substrate (~105 meV)^21^, plasmon damping through the emission of optical phonon has negligible effect^3,6,12,22,23^. Therefore, the plausible pathway for the plasmon damping is by generation of hot carriers via edge scattering-assisted Landau damping^6,24^ and resistive loss due to electron-impurity and electron-acoustic phonon scatterings^9,19,25^.

Multiple factors now contribute to the asymmetric environment within the graphene channel that determine the effective thermoelectric response of this complex system when irradiated with infrared light. First, there exists the photo-thermoelectric effect originating from the intrinsic Seebeck coefficient of graphene (*S*_1_)^2–4,17^. Second, the half-patterned graphene channel can be treated as a region consisting of two series connected thermoelectric materials with different Seebeck coefficients (Fig. 1c) for the unpatterned (*S*_1_) and patterned (*S*_2_) sections which drives the system further into thermoelectric imbalance. The different Seebeck coefficients of the two sides induce a directional photo-thermoelectric current accompanied by the bias current. The resulting potential gradient can be expressed as a function of the channel width, *X*_L_ − *X*_R_

1 $$V_S = \mathop {\int}\limits_{X_L}^{X_R} {S(x)} \frac{{\partial T_{cr}(x)}}{{\partial x}}dx$$

where *X*_L_ and *X*_R_ are the positions of the left and right contacts, respectively and *T*_cr_ is the local carrier temperature. Finally, the different carrier mobilities of the patterned and unpatterned sections of the channel lead to differential Joule heating during carrier transport, which further enhances the thermal gradient in the system by increasing the temperature-dependent Seebeck coefficients in the patterned and unpatterned regions.

Taking the above factors into consideration, finite element modelling (FEM) (Fig. 1d) was done at 295 K using COMSOL that revealed that a net temperature difference of Δ*T* ~ 4.7 K (for the incident power of 153 nW) where the patterned section has elevated temperature as can be observed in Fig. 1d. This Δ*T* across the channel yielded a photo-thermoelectric voltage *V*_PTE_ of ~0.44 mV. During the simulation, the Fermi level of graphene was maintained at −0.85 eV, and a bias voltage *V*_SD_ = 0.9 V was applied (Supplementary Figs. 9 and 10). Due to the biasing, both plasmon-assisted thermoelectric and bolometric effects contribute to the resultant D.C response of the graphene channel. In order to probe only the photo-thermoelectric voltage (*V*_PTE_), a measurement strategy was employed that eliminated bolometric effects from the signal (Supplementary Methods and Supplementary Fig. 11).

Figure 1d shows the profile of Δ*T* and *V*_PTE_ obtained across the graphene channel. It is to be stressed that generation of such a high position-dependent Δ*T* at room temperature exceeds previously reported values by an order of magnitude^1,2,4^. A series of FEM simulations were performed to investigate the role of Fermi energy and bias voltage on the photo-thermoelectric effect (Fig. 1e). It can be seen that by increasing the bias voltage, the hot-carriers transport enhances significantly such that *V*_PTE_ increases as a result of efficient carrier collection at the electrodes. Interestingly, for any bias voltage, as the Fermi energy of the graphene channel is increased from −0.55 eV to −0.8 eV, the photo-thermoelectric voltage increases and then decreases for higher Fermi energies. As mentioned earlier, increase in the Fermi energy enhances the light absorption due to the increase in available states for intraband transition which enhances hot-carrier generation, or Δ*T*, and also increases the electrical conductivity of graphene, which increases the Seebeck voltage *V*_PTE_; however, the resulting *V*_PTE_ is also a function of Seebeck coefficient (*V*_PTE_ = *S*(*E*_F_) Δ*T*) which monotonically decreases with *E*_F_. Considering these competing effects, there is a trade-off where above a threshold Fermi energy (~−0.8 eV), the effect of enhanced Δ*T* on *V*_PTE_ is negatively impacted by the lower Seebeck coefficient that results in a decrease in *V*_PTE_. Therefore, there is an optimum range of gate (*V*_G_) and bias voltage (*V*_SD_) for the best performance of the detector associated with maximum *V*_PTE_ at a desired spectral wavelength which can be observed from Fig. 1e. The predictions from FEM simulations were validated by conducting experimental measurements to quantify the photo-thermoelectric voltage generated as a function of bias and gate voltages (Fig. 1f). Photoresponse of the detector was measured by illuminating the active area with a broadband light source. An optical bandpass filter is used to eliminate other wavelengths outside of the 8–12 µm band from the broadband incident light. The experimental curves are in excellent agreement with the simulated results, which confirms that the measured signal arises from the Seebeck effect.

### D.C photoresponse

**Fig. 2:**
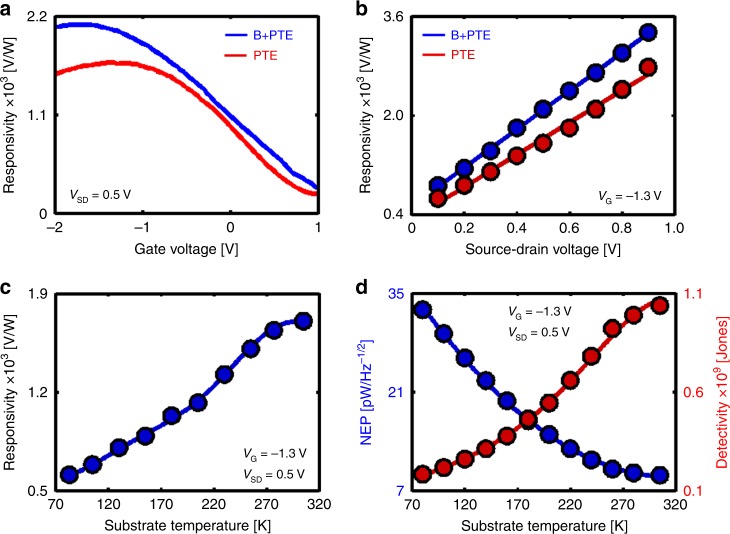
D.C photoresponse results. **a** D.C responsivity from photo-thermoelectric (red) and combined photo-thermoelectric and bolometric (blue) effects as a function of gate voltage at room temperature and *V*_SD_ = 0.5 V. **b** D.C responsivity as a function of bias voltage for gate voltage, *V*_G_ = −1.3 V. **c** D.C responsivity as a function of substrate temperature for gate voltage, *V*_G_ = −1.3 V. **d** The noise equivalent power (NEP) and specific detectivity (D*) of the half-patterned graphene detector as a function of the substrate temperature. (B: bolometric—PTE: photo-thermoelectric)

The room temperature D.C performance of the detector was characterized by the responsivity (ℛ_D.C_ = *V*_PTE_ *P*_inc_^−1^) for different bias voltages (*V*_SD_) and substrate temperatures (*T*_S_), where *P*_inc_ is the band limited incident IR power. The highest responsivity measured in the present work is 2.9 × 10^3^ VW^−1^ (Supplementary Fig. 12), which is over two orders of magnitude larger than the previous reports^4^. Figure 2a shows the combined bolometric and the photo-thermoelectric response where the total responsivity increases by 30% above the photo-thermoelectric effect alone. The responsivity follows the same trend as *V*_PTE_ and scales linearly with the applied bias voltage for both thermoelectric and combined thermoelectric-bolometric signals (Fig. 2b and Supplementary Fig. 14). Next, the effect of ambient temperature on the photoresponse of the detector was investigated, where ℛ (*V*_G_) curves were recorded at regular temperature intervals within the range of 80–320 K. As the temperature is decreased, the responsivity reduces as shown in Fig. 2c (Supplementary Fig. 13). This is in contrast to the bolometric devices where cooling improves responsivity^2,26,27^. In the proposed device, the absorption of light is almost independent of temperature, in other words, the hot-carrier generation and subsequent development of Δ*T* remains unaffected by the temperature of the sample. However, the Seebeck coefficient of graphene decreases as the temperature is lowered (Supplementary Fig. 4); consequently, for the same Δ*T*, the magnitude of *V*_PTE_ decreases gradually from 320 K to 80 K. Therefore, the responsivity of the detector decreases as the temperature is lowered which provides additional evidence that Seebeck effect is indeed the dominant phenomenon in the present detection scheme.

Noise equivalent power (NEP) is calculated by measuring the noise spectral density (*S*_n_) and the responsivity (NEP = *S*_n_ ℛ^−1^) of the detector for different substrate temperatures (Fig. 2d and Supplementary Fig. 18). Typically, thermal detectors suffer from high NEP at room temperature which is why they are cooled for better performance^26^. In contrast, as shown in Fig. 2d the proposed detector exhibits the lowest NEP at room temperature and an increase in NEP is observed upon lowering the substrate temperature. The NEP of the present detector (~7 pW.Hz^−1/2^) is more than one order of magnitude lower than thermal photodetectors^2,26^ which establishes its superiority as an uncooled, room-temperature mid-IR photodetector. The specific detectivity (D*) is derived from the NEP and the detector active area (A) as $D^ \ast = \sqrt A \,NEP^{ - 1}\left[ {cm\,\sqrt {HZ} \,W^{ - 1}} \right]$ or [Jones]). The maximum D* at E_F_ = −0.8 eV and V_SD_ = 0.9 V is measured to be 1.1 × 10^9^ Jones (Fig. 2d) which clearly outperforms all graphene-based MIR photodetectors reported till date^1,2,4,5,7,26–35^.

### A.C photoresponse

**Fig. 3:**
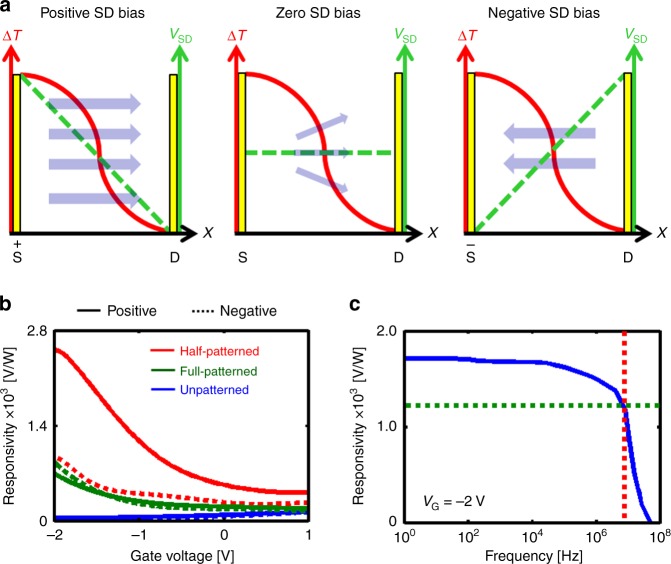
A.C photoresponse results. **a** Schematic showing the A.C photocurrent for different measurement conditions. **b** A.C responsivity of the half-patterned (red), full-patterned (green) and unpatterned (blue) graphene detectors for the positive (solid) and negative (dashed) offsets (0.25 V) at *f* = 20 Hz. **c** A.C responsivity as a function of frequency for *V*_G_ = −2V. The crosshair shown in dashed lines corresponds to the cut-off frequency

To further elucidate the role of LSPs in hot carrier generation and how the proposed asymmetric design excels in creating a high responsivity detector, we compare the A.C photoresponse of three detectors that were fabricated with (i) half-patterned, (ii) full-patterned and (iii) unpatterned graphene channels, respectively. We postulate that for the unpatterned and full-patterned detectors, the photoresponse primarily arises from the bolometric effect. Furthermore, owing to the symmetric design of the unpatterned and full-patterned devices, it is expected that the polarity of bias voltage should not affect the photoresponse. In contrast, due to the asymmetric architecture of the half-patterned detector, a bias voltage in the direction of Δ*T* favors the collection of hot-carriers compared to the opposite bias. For the zero-bias condition, the asymmetric case is expected to yield a finite photoresponse, however, the symmetric cases should result in zero photoresponse owing to omnidirectional scattering of hot carriers. Figure 3a schematically illustrates hypotheses that were tested by the following measurements. The experimentally measured responsivity at *f* = 20 Hz shown in Fig. 3b confirms the working hypothesis. The full-patterned device exhibited higher responsivity than the unpatterned device, which is attributed to the enhanced infrared absorption. On the other hand, the half-patterned device showed significantly improved responsivity arising from higher Δ*T* across the graphene channel. In addition, it can be seen that the polarity of bias voltage has significant effect on the responsivity of the half-patterned device unlike the symmetric full-patterned and unpatterned devices. The positive bias condition (source voltage: 0 V to +0.5 V) assists the drift of the hot-carriers (holes) generated on the patterned section towards the drain (ground), whereas in the negative bias condition (source voltage: 0 V to −0.5 V), the flow of holes towards the drain (ground) is inhibited leading to diminished responsivity, as shown in Fig. 3b.

The time response of the half-patterned detector was measured to quantify the operational bandwidth. Due to the ultrafast plasmonic excitation and charge transport in graphene, a fast photoresponse is expected^6^. Since light modulation by mechanical chopping was not a feasible technique for high-speed measurements, we adopted an alternate method to study the A.C photoresponse by electronic modulation of the source-drain bias (Supplementary Fig. 15) from 200 Hz to 100 MHz. The corresponding A.C responsivity as a function of frequency is shown in Fig. 3c. We observe a constant responsivity up to 8 MHz corresponding to a 3 dB cut-off time constant of *τ*_res_ = 125 ns. It is to be noted that the measured time constant is larger than the Dirac plasmon lifetime (~10^−15^ s) and is limited by the capacitance of the external circuitry^36^.

**Fig. 4:**
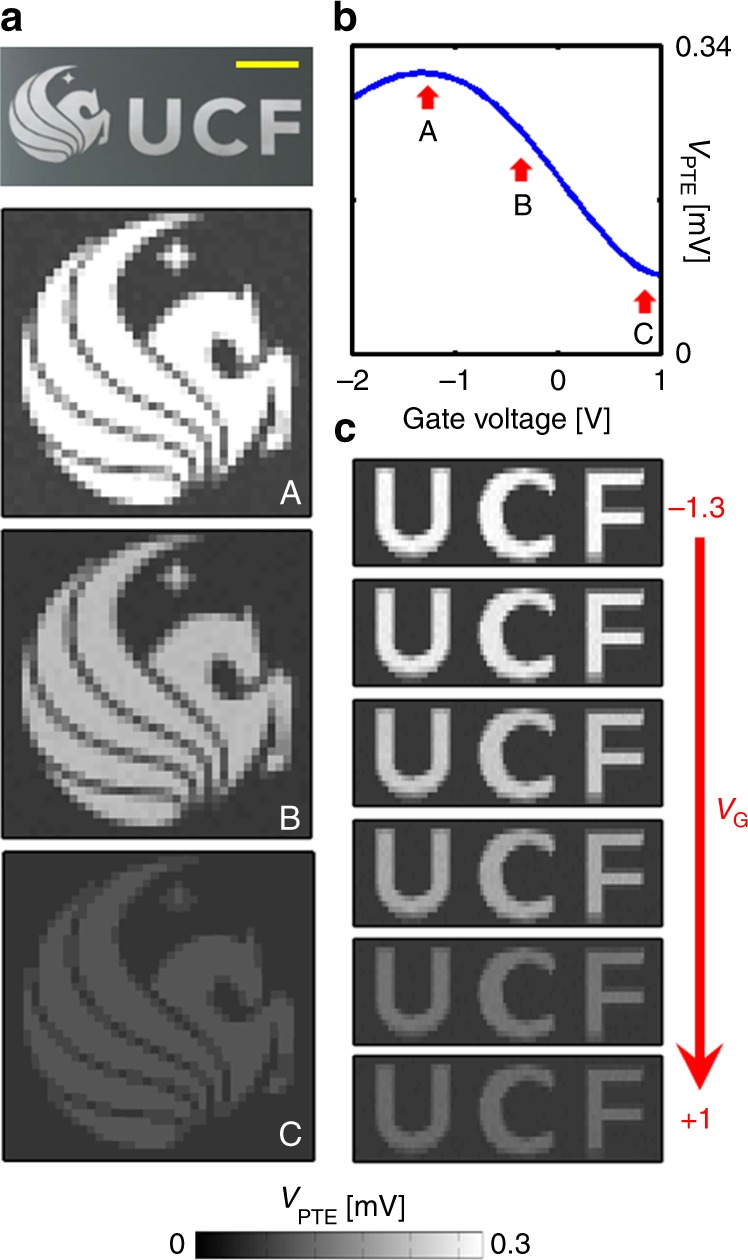
Infrared imaging. The single-pixel imaging results, **a**, **c** for different gate voltages. The contrasts of the images scale according to the photo-thermoelectric voltage as a function of gate voltage, **b**. Figure (**a**-top) is the object used for imaging. The yellow scale bar shows 5 mm

The proposed asymmetric graphene device is a multispectral gate tunable infrared detector. This opens up the possibility for making an uncooled multi-pixel infrared camera with performance comparable to the commercial cooled cameras. To demonstrate the real performance of the photodetector, a single-pixel imaging method (Supplementary Fig. 17)^37^ was used to image a Pegasus and UCF logo printed on a substrate. The tunable response of the detector is evident from greyscale images shown in Fig. 4 and, also Supplementary Movies 1 and 2 for different gate voltages.

## Discussion

In conclusion, this work demonstrates outstanding room temperature photodetection using 2D monolayer graphene that is possible by the interplay between multiple physical phenomena: (i) tunable enhanced infrared absorption induced by localized Dirac plasmonic excitations, (ii) graphene mobility engineering, and (iii) excitation of asymmetric hot carriers and consequent electronic photo-thermoelectric effect. The asymmetric graphene channel design facilitates generation of high-temperature gradient (Δ*T* ~ 4.7 K, *P*_in_ = 153 nW) which is critical to the remarkable photoresponse. We identify various processes contributing to the photoresponse and present conclusive evidence that the fast (τ_res_ ~ 100 ns), high responsivity (2900 VW^−1^) and high D* ~ 1.1 × 10^9^ Jones can be attributed to the photo-thermoelectric effect. The proposed frequency-tunable graphene detectors not only offer spectroscopic detection but also pave the path towards dynamic multispectral imaging in the IR domain, which is lacking in the present IR imaging technologies.

## Methods

### Fabrication

The large-scale monolayer graphene grown by chemical vapor deposition (CVD) method on copper foil was transferred on the Si^++^ (100 μm)/Al_2_O_3_ (8 nm) substrate. The source and drain contacts were fabricated by UV-lithography following by Ti/Au (3 nm/60 nm) deposition. The electron beam lithography (EBL) following by oxygen-plasma etching techniques was used to nanopattern the half side of transferred graphene with period *P* = 600 nm and diameter *D* = 400 nm.

### D.C photo-thermoelectric voltage measurement

For a fixed gate voltage, a D.C bias voltage (*V*_SD_) was applied across the source-drain (SD) terminals and the resulting current *I*_1_ = *I* + *I*_TE_ and *I*_2_ = –*I* + *I*_TE_ were measured for applied voltage ± *V*_SD_, where *I* is the current generated by the bias voltage and *I*_TE_ is the thermoelectric current (*I*_TE_ = 0.5 × (*I*_1_ + *I*_2_)). This thermoelectric current was measured in dark (*I*_TE-D_) and in the presence of mid-IR light (*I*_TE-L_). Any contribution due to the photoconductive effect is expected to be independent of the polarity of applied bias voltage, which was thereby eliminated in the *I*_TE-L_ calculation. Therefore, the photo-thermoelectric current and voltage can be calculated as *I*_PTE_ = *I*_TE-L_ − *I*_TE-D_ and, *V*_PTE_ = *R*_G_*I*_PTE_ respectively (Supplementary Methods). The D.C responsivity (ℛ_D.C_ = *V*_PTE_ *P*_inc_^−1^) was calculated by using the measured incident light power (*P*_inc_), the gate-tunable graphene resistance (*R*_G_) (Supplementary Fig. 19) and *I*_PTE_. The circuit diagram is shown in Supplementary Fig. 11.

### A.C photoresponse measurement

The circuit diagram for A.C photoresponse is shown in Supplementary Fig. 15. In the measurement setup, the sample was placed in front of a broadband blackbody source with a 8–12 µm filter in between. The Fermi level of graphene was fixed by applying a constant gate voltage. First, a sinusoidal bias voltage with a positive offset of 0.25 V was applied in dark to the source-drain terminals. Due to this, an A.C voltage was developed across resistor *R*_2_ that was at the same frequency as the input bias voltage $\left[ {V_{SD + }^{dark} = 0.25 + \sin (2\pi f)} \right]$. The voltage across *R*_2_ was recorded using a lock-in-amplifier. Next, in the presence of light, the voltage across *R*_2_ $\left[ {V_{SD + }^{light}} \right]$ was measured. The A.C photovoltage was calculated by taking the difference $\left[ {V_{SD + }^{PV} = V_{SD + }^{light} - V_{SD + }^{dark}} \right]$. The term $V_{SD + }^{PV}$ includes photoresponse from both photo-thermoelectric and photoconductive effects. Therefore, similar to the D.C responsivity measurement protocol, in order to eliminate the photoconductive effect the A.C photovoltage $(V_{SD - }^{PV})$ was measured for a negative offset bias voltage and the difference $V_{SD + }^{PV} - V_{SD - }^{PV}$ yields the A.C photo-thermoelectric voltage which is plotted in Supplementary Fig. 16.

### FEM simulation

The COMSOL Multiphysics 5.3a software was used to simulate the performance of the detector. The overall goal of simulations was to find the time-dependent solution for the bias-dependent photo-thermoelectric current, which was further used to calculate the photo-thermoelectric voltage (*V*_PTE_) and the responsivity *R* = *V*_PTE_ *P*_inc_^−1^. The built-in modules Electric Currents and Heat Transfer in Solids coupled with the multiphysics module Thermoelectric Effect were applied to predict the behavior of the detector.

The sample geometry in the simulations was identical to the real detector except for the length of the simulated detector, which was decreased to 20 µm as compared to 200 µm in the experiment, in order to reduce the computation time. The simulated detector was 20 µm wide (contacts and graphene) and 20 µm long. The channel width of detector was 10 µm wide and 20 µm long, where half of the width of the graphene sheet was patterned, and the other half kept unpatterned. The gold terminals were 5 µm by 20 µm, and the thicknesses of graphene, gold contacts, aluminum oxide, and silicon were 0.5 nm, 50 nm, 8 nm, and 3 µm, respectively. Gold, Silicon, and Aluminum oxide materials were directly imported from COMSOL material library, while the experimentally measured parameters were used for graphene. The electrical conductivity and Seebeck coefficient were gate-dependent for graphene, measured experimentally for the patterned and unpatterned graphene, separately. The temperature-independent electrical conductivity was used for all materials to neglect the bolometric effects.

The bias voltage was applied across the gold terminals; one side was set to ground, and the other at high potential. Except gold terminals and graphene, everything was considered electrically insulated. The current conservation boundary condition was applied for the whole geometry, and the initial values were set to *V*_0_ = 0 V. In order to add the contact resistance, the electrical contacts were introduced between gold and graphene. The heat flux was applied in the form of rectangular pulse of period 4 ms, which means for the first two milliseconds the heat flux was zero, corresponding to the dark state in the experiment. For the next two milliseconds, nonzero heat flux was applied on the patterned side of graphene using laser heating. A Gaussian beam with the spot size *R*_spot_ = 2 mm and the incident power *P*_inc_ = 153 nW was used. The absorbed heat flux depended on the absorption at different Fermi levels. The gate dependence of the light absorption was calculated by using the Lumerical FDTD software, which ranged from *A* = 34% at *E*_F_ = −0.55 eV to *A* = 60% at *E*_F_ = −1.0 eV for the patterned graphene (Supplementary Methods).

The bottom side of the detector was kept at fixed temperature using the boundary condition in the software. The initial value of the temperature was set to *T*_0_ = 293.15 K, and the boundary condition open boundary was used across all the sides of the detector, except top and bottom surfaces which means the heat can flow inside or outside across the cross-sectional boundary depending on the ambient temperature. Thermal contacts were used between graphene, aluminum oxide, and silicon to control heat transfer in the vertical direction. The free tetrahedral mesh for gold and the free triangular mesh at the graphene surface were used, which were swept in vertical direction for the remaining geometry.

The time-dependent solver with very low relative tolerance of 10^−5^ was used to measure the time-dependent thermoelectric voltage across the terminal for different Fermi energies. The dark and light thermoelectric voltages *V*_TE,D_ and *V*_TE,L_ were measured in absence and presence of the incident heat flux, respectively. The photo-thermoelectric voltage *V*_PTE_ was then calculated by subtracting the dark from the light voltage, i.e. *V*_PTE_ = *V*_TE, L_ − *V*_TE, D_.

## Supplementary information

Supplementary Information

Description of Additional Supplementary Files

Supplementary Movie 1

Supplementary Movie 2

## Data Availability

The authors declare that the main data supporting the findings of this study are available within the article and its Supplementary Information files. Extra data are available from the corresponding author upon request.
