# Supplementary material for: Dirac plasmon-assisted asymmetric hot carrier generation for room-temperature infrared detection
Source: Nat Commun. 2019 Aug 2;10:3498. doi: 10.1038/s41467-019-11458-5 (PMC6677812; doi:10.1038/s41467-019-11458-5)
Supplement: Supplementary file 1 — Supplementary Information [file 41467_2019_11458_MOESM1_ESM.pdf]

Supplementary Information for

Dirac Plasmon-Assisted Asymmetric Hot Carrier Generation for Room-Temperature Infrared Detection

Alireza Safaei[1,2ǂ], *Sayan Chandra*[2ǂ], *Muhammad Waqas Shabbir*[1,2], *Michael N. Leuenberger*[1,2,3], *Debashis Chanda*[1,2,3]*

[1]Department of Physics, University of Central Florida, Orlando, Florida 32816, USA.

[2]NanoScience Technology Center, University of Central Florida, Orlando, Florida 32816, USA.

[3]CREOL, The College of Optics and Photonics, University of Central Florida, Orlando, Florida 32816, USA.

KEYWORDS: Infrared detection, Graphene, Thermoelectric, Seebeck effect, Room temperature, light absorption, electrostatic tunability, localized surface plasmon

ǂ These authors contributed equally to this work.

Supplementary Methods

Raman spectroscopy was performed on the transferred graphene to verify if oxygen etching during the nanopatterning process altered the characteristic optical phonon peaks at ~1590 (the G peak) and ~2700 cm^{-1}. The results in Supplementary Figure 1 shows phonon peaks at ~1590 (the G peak) and ~2700 cm^{-1} associated with monolayer graphene[1]. This figure shows the Raman spectra of the pristine (unpatterned) and nanopatterned graphene which confirms the absence of graphene oxide (GO) or reduced graphene oxide (RGO).

To determine the experimental value of the Fermi energies at different gate voltages and carrier mobility, the measured resistance of graphene was fitted to the theoretical formula ($R = R_0 + 1/ne\mu$), as shown in Supplementary Figure 2. Here R_0 is the minimum resistance at V_G= -1 V, $n = C\Delta V/\mathrm{e}$ is the electron density and e is the Coulomb charge. The high-k gate-dielectric is 15 nm of Al_2O_3 with a measured capacitance of C = 0.93 μFcm^{-2} which has a high stability in time [2-4]. The Fermi energy of graphene at 0 V is found to be -0.6 eV which suggests that the graphene sheet is self-doped to be p-type. In addition, the carrier mobility is determined to be 500 $cm^2\ V^{-1}s^{-1}$ for the unpatterned channel, which decreases for the half-patterned case (350 $cm^2\ V^{-1}s^{-1}$) and the full-patterned graphene (250 $cm^2\ V^{-1}s^{-1}$), as expected for pattern-induced enhanced scattering. This clearly establishes the effect of nanopatterning on the electronic properties of graphene.

Supplementary Figure 3 shows the derivative of the measured conductivity of the graphene channel ($\left[d \ln(\sigma(E))/\, dE\right]_{E=E_\mathrm{F}}$) for the unpatterned, half-patterned and full-patterned devices. The Seebeck coefficients for different substrate temperatures was derived by using these derivatives $S = (\pi^2 k_b^2 T/3\mathrm{e}^2)\left[d \ln(\sigma(E))/\, dE\right]_{E=E_\mathrm{F}}$ [5,6], as shown in Supplementary Figure 4 which is a decreasing function of the Fermi energy. We characterize the temperature dependence

of the Seebeck coefficient for the half-patterned graphene device which is eventually used for infrared detection. Supplementary Figure 4 shows the back-gate dependence of S measured at different temperature intervals in the range of 80 K – 300 K. The temperature dependent S of the half-patterned graphene device follows a similar trend to that reported for pristine graphene [7,8], which substantiates that while the magnitude of S may have diminished as a result of nanopatterning, the thermoelectric properties of the half-patterned graphene can be understood from the framework of pristine graphene. To understand the reason of this behavior, the Seebeck coefficient of the pristine graphene was calculated in the range of E_F= -2.9eV to E_F= 0 eV, for C = 0.93 μF/cm^2 and μ = 500 cm^2 V^{-1}s^{-1}. As shown in Supplementary Figure 5, the Seebeck coefficient is a polynomial function of Fermi energy which is consistent with previous reports [7,8], and is proportional to $S \propto \frac{T}{\sqrt{n_\mathrm{h}}} \propto \frac{T}{E_\mathrm{F}}$ from E_F= -1.0 eV to E_F=-0.55 eV [9].

In Supplementary Figure 1c of the main manuscript, the Seebeck coefficient S1 (unpatterned) and S2 (patterned) for graphene are presented. The variable quantity $S(x)$ in Equation 1 of the main manuscript is defined as a step function with the values S_1 for $0 \le x \le L/2$ and S_2 for $L/2 < x \le L$ for the case of a half-patterned channel.

The Seebeck coefficient of the patterned/unpatterned graphene was calculated using the very well established Mott relation [6,10] which we briefly describe below.

The Mott relation describes Seebeck Coefficient as $S = \frac{\pi^2 k_B{}^2 T}{3e} \frac{\partial \ln \sigma}{\partial \varepsilon_F}$, where $\boldsymbol{\sigma}, k_B$, e, and $\boldsymbol{\varepsilon}_\mathrm{F}$ are electrical conductivity, Boltzmann constant, Coulomb charge, and Fermi energy, respectively [6-8], obtained from the non-interacting model which is valid for $k_B T \ll E_F$. The electrical conductivity

was extracted from the source-drain current I_{sd} via $\sigma = I_{sd}\dfrac{L}{WV_{sd}}$, where L and W are the channel length and width, respectively, and V_{sd} is the drain-source voltage (Supplementary Figure 2). It means we can write S as $S = \dfrac{\pi^2 k_B{}^2 T}{3e}\dfrac{1}{R}\dfrac{dR}{dV_g}\dfrac{dV_g}{dE}\bigg|_{E=E_F}$, where R is the resistance as a function of gate voltage V_g [6]. In the FEM model, the half-patterned graphene channel was treated as a region consisting of two series connected thermoelectric materials with different gate-dependent Seebeck coefficients for the unpatterned (S_1) and patterned (S_2) sections. The respective Seebeck Coefficients for the patterned and unpatterned regions were fed into the FEM thermoelectric module while computing Supplementary Equation 6. Consequently, the COMSOL simulations yielded temperature and potential profiles which are presented in Figure. 1d of the manuscript.

Finite difference time domain (FDTD) simulations were performed over different periods and hole diameters to maximize the infrared absorption in the 8 – 12 μm band as shown in Supplementary Figure 6. According to the results, the maximum light absorption happens for the hexagonal array in graphene with period P = 600 nm and hole diameter D = 400 nm.

Illuminating light on the cavity-coupled graphene with hexagonal array of nanohole excites LSP and confines the field on the edges of the nanoholes to generate hot-carriers[2,11-16]. The light absorption and LSPR frequency are independent of the polarization and the incident angle of light (θ_{i}) for $\theta_{\mathrm{i}} \leq 50^{\circ}$ [2]. The gate-tunable light absorption spectra of the cavity coupled nanopatterned graphene is electrostatically tunable ($\Delta\boldsymbol{\lambda}_{\mathrm{res}}$= 2.5 μm) with maximum value of ~ 60%, as shown in Supplementary Figure 7a along with the absorption of a cavity-coupled unpatterned graphene. The extraordinary near-field enhancement by a factor of 500 (Supplementary Figure 7b) for nanohole highlights the extraordinary light-matter interaction. Supplementary Figure 8a shows the silicon

substrate has ~70% light transmission in the desired spectrum window (8-12 μm). The leakage current of the gate-dielectric is an important factor in power usage which is very low (~10^{-11} A) for 15 nm thick layer of the grown Al_2O_3, as shown in Supplementary Figure 8b.

The maximum value of the bias voltage is limited by the breakdown current density of graphene[17] which in this case is 12 Acm^{-2}, hence, 0.9 V was chosen as the upper limit for the bias voltage for an active detector area of 10x200 μm^2.

The COMSOL Multiphysics 5.3a software was used to simulate the performance of the detector. The overall goal of simulations was to find the time dependent solution for bias-dependent photothermoelectric voltage, which was further used to calculate the photothermoelectric voltage (V_{PTE}) and the responsivity $R=V_{\mathrm{PTE}}/P_{\mathrm{inc}}$, where P_{inc} is the power of the incident light. The built-in modules "Electric Currents" and "Heat Transfer in Solids" coupled with the multiphysics module "Thermoelectric Effect" were used to simulate the photothermoelectric process in the graphene detector. There are different coupled equations in these modules to solve and find the electric potential (V) and temperature (T):

$$\mathbf{E} = -\nabla V, \tag{1}$$

$$\nabla.\mathbf{J} = 0, \tag{2}$$

$$\mathbf{J} = \sigma\mathbf{E} + \frac{\partial \mathbf{D}}{\partial t} + \mathbf{J}_e, \tag{3}$$

$$\rho c_p \frac{\partial}{\partial t} T + \rho c_p \mathbf{u}.\nabla T - \nabla.\mathbf{q} = Q, \tag{4}$$

$$\mathbf{q} = -k\nabla T, \tag{5}$$

$$\prod = ST, \quad \mathbf{q} = \prod \mathbf{J}, \quad \text{and} \quad \mathbf{J}_e = -\sigma S \nabla T, \tag{6}$$

where $\boldsymbol{J}$ is the current density, $\boldsymbol{E}$ is the electric field, $\boldsymbol{D}$ is the displacement field, S is the Seebeck coefficient, and $\prod$ is the Peltier coefficient. $\mathbf{J}_e$ is an external current density, contributed by the generated hot electrons. The other constants $\boldsymbol{\sigma}, \boldsymbol{\rho}$, c_p, and k represent the electrical conductivity, the mass density, the specific heat capacity at constant pressure, and the thermal conductivity, respectively. Moreover, $\mathbf{q}$ is the conductive heat flux, Q is the heat source (or sink), and $\mathbf{u}$ is the velocity field defined by the translational motion subnode when parts of the model are moving in the material frame.

The sample geometry in the simulated device was identical to the real detector except for the length, which was decreased to 20µm as compared to 200µm in the experiment, in order to reduce the computation time. The channel width of detector was 10µm wide and 20µm long, where half of the width of the graphene sheet was patterned, and the other half was unpatterned. The gold terminals were 5µm by 10µm, and the thicknesses of graphene, gold contacts, aluminum oxide, and silicon, were 0.5nm, 50nm, 15nm, and 3µm, respectively. Gold, Silicon, and Aluminum oxide materials were directly imported from COMSOL material library, while the experimentally measured parameters were used for graphene. As explained in detail in the manuscript, the Seebeck coefficient was calculated by Mott's approximation $S = \frac{\pi^2 k_B{}^2 T}{3e} \frac{\partial \ln \sigma}{\partial \varepsilon_F}$, where $\boldsymbol{\sigma}, k_B$, e, and $\boldsymbol{\varepsilon}_F$ are electrical conductivity, Boltzmann constant, Coulomb charge, and Fermi energy, respectively. The electrical conductivity and Seebeck coefficient were gate-dependent for graphene, and electrical conductivity and Seebeck coefficient were measured experimentally for patterned graphene and unpatterned graphene. The heat capacity of graphene at room temperature was set to c_p = *700 J/(kg-K)*. To avoid the bolometric effect, the temperature-independent electrical conductivities were used for all the materials.

The bias voltage was applied across the gold terminals; one side was set to ground, and the other one at high potential. Except the gold terminals and graphene, everything else was electrically insulated. In addition, the current conservation boundary condition was applied for the whole geometry, and the initial values were set to V=0 V. In order to add contact resistance similar to the fabricated device, electrical contacts were introduced between gold. The heat flux was applied in form of rectangular pulse with the period of 4 ms, which means for the first two milliseconds there was zero heat flux, corresponding to the dark state in the experiment, while for the next two milliseconds nonzero heat flux was applied on the patterned side of graphene using a laser heating. The Gaussian beam with spot size R_{spot} = 2 mm and incident power P_{inc}=153 nW was used as the incident power which set the heat flux $q_0 = (2P_{Incident}/\pi R^2{}_{spot})\exp(-2R^2{}_{focus}/R^2{}_{spot})$. The absorbed heat flux depends on the light absorption at different Fermi levels. The light absorption as a function of gate voltage was calculated by Lumerical FDTD software, which ranged from A=34% *at* E_{F} = -0.55 eV to A=60% *at* E_{F} = -1.0 eV for patterned graphene. It means the absorbed heat flux was $\mathrm{q}_{\mathrm{absorbed}} = Aq_0$.

The bottom side of the detector was kept at fixed temperature by using the boundary condition “temperature” in the software. The initial value of the temperature was set to T_0=293.15 K, and the boundary condition “open boundary” was used across all the sides of the detector, except top and bottom surfaces, which means heat could flow inside or outside across the cross-sectional boundary depending on the ambient temperature. Thermal contacts were used between graphene, aluminum oxide, and silicon to control the heat transfer in the vertical direction. A user-controlled mesh, namely the free tetrahedral mesh was used for gold, while the free triangular mesh was applied at the graphene surface, which were swept in vertical direction for the remaining geometry.

The time dependent solver with very low relative tolerance of 10^{-5} was used to measure the time dependent thermoelectric current by passing through the terminal for different Fermi levels. The dark and light thermoelectric voltages $V_{TE,D}$ and $V_{TE,L}$ were measured in absence and presence of the incident heat flux, respectively. The photothermoelectric voltage V_{PTE} current was then calculated by subtracting the dark from the light current, i.e.

$$V_{PTE} = V_{TE,\,L} - V_{TE,\,D}\,. \qquad (7)$$

Supplementary Figures 9 and 10 show the 3D and 2D cross-section profiles of the generated temperature and photovoltage.

For a fixed gate voltage, a D.C bias voltage (V_{SD}) was applied across the source-drain (SD) terminals and the resulting current $I_1 = I + I_{TE}$ and $I_2 = –I + I_{TE}$ were measured for applied voltage $\pm V_{SD}$, where I is the current generated by the bias voltage and I_{TE} is the thermoelectric current ($I_{TE}=(I_1+I_2)/2$). This thermoelectric current was measured in dark ($I_{TE\text{-}D}$) and in the presence of mid-IR light ($I_{TE\text{-}L}$). Any contribution due the photoconductive effect is expected to be independent of the polarity of applied bias voltage, which was thereby eliminated in the $I_{TE\text{-}L}$ calculation. Therefore, the photothermoelectric current and voltage can be calculated as $I_{PTE}= I_{TE\text{-}L}-I_{TE\text{-}D}$ and, $V_{PTE} = R_G I_{PTE}$ respectively. The D.C responsivity ($\mathcal{R}_{D.C} = V_{PTE} / P_{inc}$) was calculated by using the measured incident light power (P_{inc}), the gate-tunable graphene resistance (R_G) and I_{PTE}. The circuit diagram is shown in Supplementary Figure 11.

As mentioned in the manuscript, applying a D.C bias as the source-drain voltage helps to increase the drift velocity of the hot carriers. In addition, asymmetric joule heating from the local photo-induced current helps to enhance the thermoelectric signal. It means the larger bias, the larger responsivity is and as long as the bias voltage does not lead to a dielectric breakdown,

increase in the bias can assist in detection. Supplementary Figure 12a shows the simulated D.C responsivity for different bias voltages which is in very good agreement with the experimental results in Supplementary Figure 12b.

According to Supplementary Figure 4, decrease in the graphene temperature lowers the Seebeck coefficient. One way to prove the output signal is due to photothermoelectric effect is to study the behavior of the generated voltage at different substrate temperatures. The simulated results (Supplementary Figure 13a) has very good agreement with the measured responsivities (Supplementary Figure 13b) which show that as the temperature is decreased, the responsivity reduces which provides additional evidence that the Seebeck effect is indeed the dominant phenomenon in the present detection scheme. According to Supplementary Figure 14, the responsivity scales linearly with the applied bias voltage for both thermoelectric and combined thermoelectric-bolometric signals.

The circuit diagram for A.C photoresponse is shown in Supplementary Figure 15. In the measurement setup the sample was placed in front of a broadband blackbody source with a $\boldsymbol{\lambda}$= 8 – 12 µm filter in between. The Fermi level of graphene was fixed by applying a constant gate voltage. First, a sinusoidal bias voltage with a positive offset of 0.25 V was applied in dark to the source – drain terminals. Due to this, an A.C voltage was developed across resistor R_2 that was at the same frequency as the input bias voltage [$V_{SD+}^{dark} = 0.25 + \sin(2\pi f)$]. The voltage across R_2 was recorded using a lock-in-amplifier. Next, in the presence of light, the voltage across R_2 [$V_{\mathrm{SD+}}^{\mathrm{light}}$] was measured. The A.C photovoltage was calculated by taking the difference [$V_{\mathrm{SD+}}^{\mathrm{PV}} = V_{\mathrm{SD+}}^{\mathrm{light}} - V_{\mathrm{SD+}}^{\mathrm{dark}}$]. The term $V_{\mathrm{SD+}}^{\mathrm{PV}}$ includes photoresponse from both photothermoelectric and photoconductive effects. Therefore, similar to the D.C responsivity measurement protocol, in order

to eliminate the photoconductive effect the A.C photovoltage ($V_{\mathrm{SD}-}^{\mathrm{PV}}$) was measured for a negative offset bias voltage and the difference $V_{\mathrm{SD}+}^{\mathrm{PV}} - V_{\mathrm{SD}-}^{\mathrm{PV}}$ yields the A.C photothermoelectic voltage.

To further elucidate the role of LSPs in hot carrier generation and how the proposed asymmetric design excels in creating a high responsivity detector, we compare the A.C photoresponse of three detectors that were fabricated with (i) half-patterned, (ii) full-patterned and (iii) unpatterned graphene channels, respectively. We postulate that for the unpatterned and full-patterned detectors, the photoresponse primarily arises from the bolometric effect. Furthermore, owing to the symmetric design of the unpatterned and full-patterned devices, it is expected that the polarity of bias voltage should not affect the photoresponse. In contrast, due to the asymmetric architecture of the half-patterned detector, a bias voltage in the direction of ΔT favors the collection of hot-carriers compared to the opposite bias. For the zero-bias condition, the asymmetric case is expected to yield a finite photoresponse, however, the symmetric cases should result in zero photoresponse owing to omnidirectional scattering of hot carriers. Supplementary Figure 16a schematically illustrates hypotheses that were tested by the following measurements. The experimentally measured responsivity at f = 20 Hz shown in Supplementary Figure 16b confirms the working hypothesis. The full-patterned device exhibited higher responsivity than the unpatterened device, which is attributed to the enhanced infrared absorption. On the other hand, the half-patterned device showed significantly improved responsivity arising from higher ΔT across the graphene channel. In addition, it can be seen that the polarity of bias voltage has significant effect on the responsivity of the half-patterned device unlike the symmetric full-patterned and unpatterned devices. As discussed earlier, the positive bias condition (source voltage: 0 V to 0.5 V) assists the drift of the hot-carriers (holes) generated on the patterned section towards the drain (ground), whereas in the negative bias condition (source voltage: 0 V to -0.5 V),

the flow of holes towards the drain (ground) is inhibited leading to diminished responsivity as shown in Supplementary Figure 16b.

To demonstrate the real performance of the photodetector, a single-pixel imaging method [18] was used to image a Pegasus and UCF logo printed on a substrate. A quantum cascade laser (QCL), an automatic motor stage connected to the object (stencil), and two objective lenses were used for the imaging by the single-pixel graphene detector, as shown in Supplementary Figure 17.

The noise spectral density is measured for different circuit frequency by using the same circuit used to characterize the AC responsivity of the detector. The frequency was applied by an external function generator and the noise spectral density (S_n) was measured directly via a Locked-In Amplifier in the 10 Hz to 1 MHz bandwidth which is below the cut-off frequency of the detector. Supplementary Figure 18a shows the noise spectral density at room temperature at E_F= -1 eV. Since the responsivity below the cut-off frequency is constant (Figure. 3c of the main manuscript), the noise equivalent power (NEP) was calculated by taking the average of the noise spectral density (S_n) over the measured spectral range and normalized by the responsivity (NEP = S_n /$\mathcal{R}$) [19]. Decrease in temperature lowers S_n, as shown in Supplementary Figure 18b. In Supplementary Figure 18c, we show that S_n decreases as p-doping in graphene is increases. As seen from Figure. 2c of the main manuscript, changing the temperature has much higher impact on the responsivity compared to Sn (Supplementary Figure 18b) and because of that, NEP is a decreasing and detectivity ($D^* = \sqrt{\mathrm{A}}/\mathrm{NEP}$) is an increasing function of the substrate temperature, as shown in Supplementary Figure 18d. Moreover, as shown in Supplementary Figure 18e, NEP increases and detectivity decreases as the Fermi energy of graphene is electrostatically doped from -0.5 eV to -1 eV.

In this fabrication, the CVD grown graphene is highly p-doped (self-doped to about -0.55 eV) due to the polymer residue (PMMA) from the transfer process which restricted the operation in the p-doped region, away from the CNP, as depicted in the analytically derived electrical resistance in Supplementary Figure 19.

Supplementary Figures

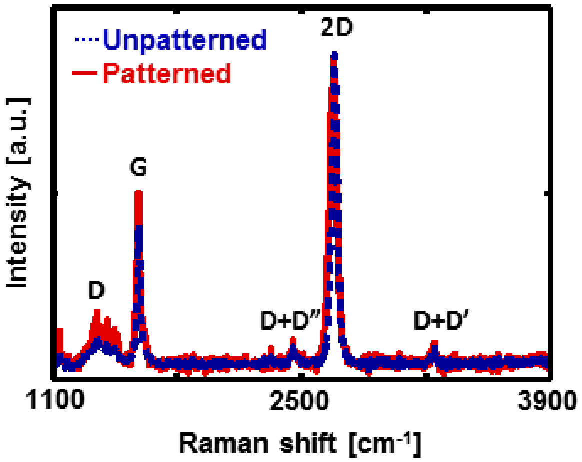

Supplementary Figure 1| The Raman spectroscopy. The Raman spectroscopy of the pristine and patterned monolayer graphene (E_F= -0.6 eV).

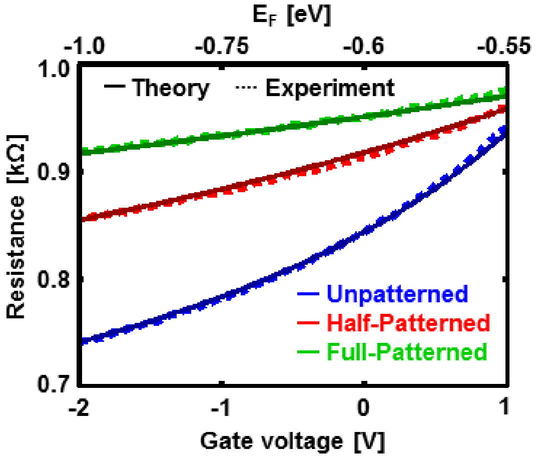

Supplementary Figure 2| Electrostatic doping of graphene. The experimental and analytical model diagrams are overlaid to find the carrier mobility of the unpatterned, half-patterned and full-patterned graphene sheets.

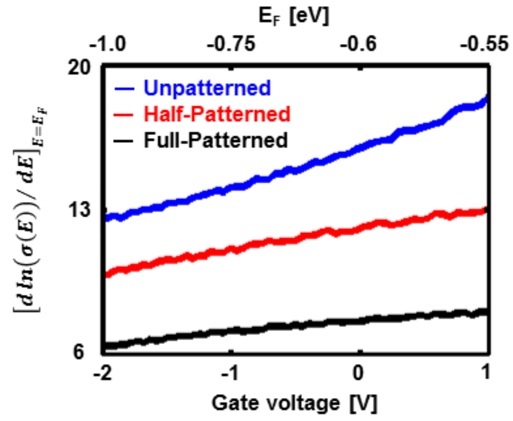

Supplementary Figure 3| The derivative of the conductivity of the graphene channel from the experimentally measured curves.

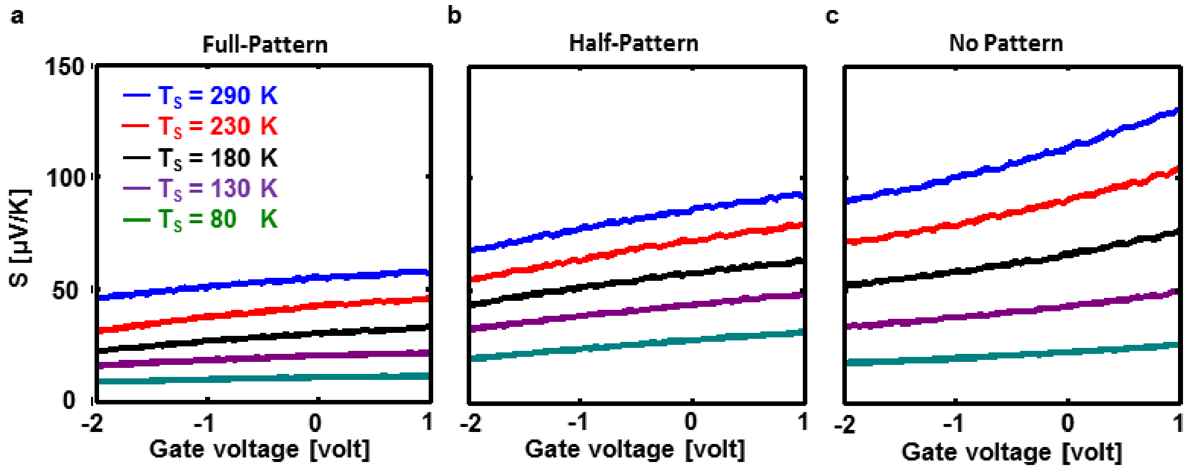

Supplementary Figure 4| Seebeck coefficient. Seebeck coefficient of Full-pattern (a), half-patterned (b), and unpatterned (c) graphene samples as a function of gate voltage for different substrate temperatures.

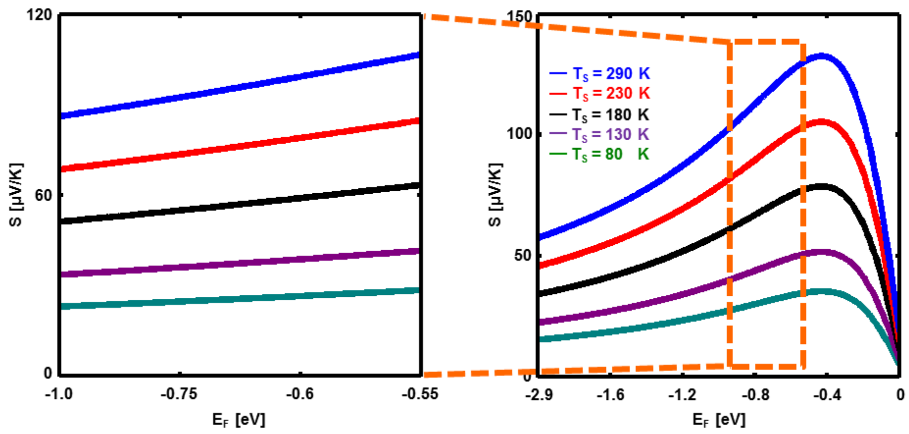

Supplementary Figure 5| Theoretical Seebeck coefficient. Seebeck coefficient of Full-pattern graphene samples as a function of gate voltage for different substrate temperatures.

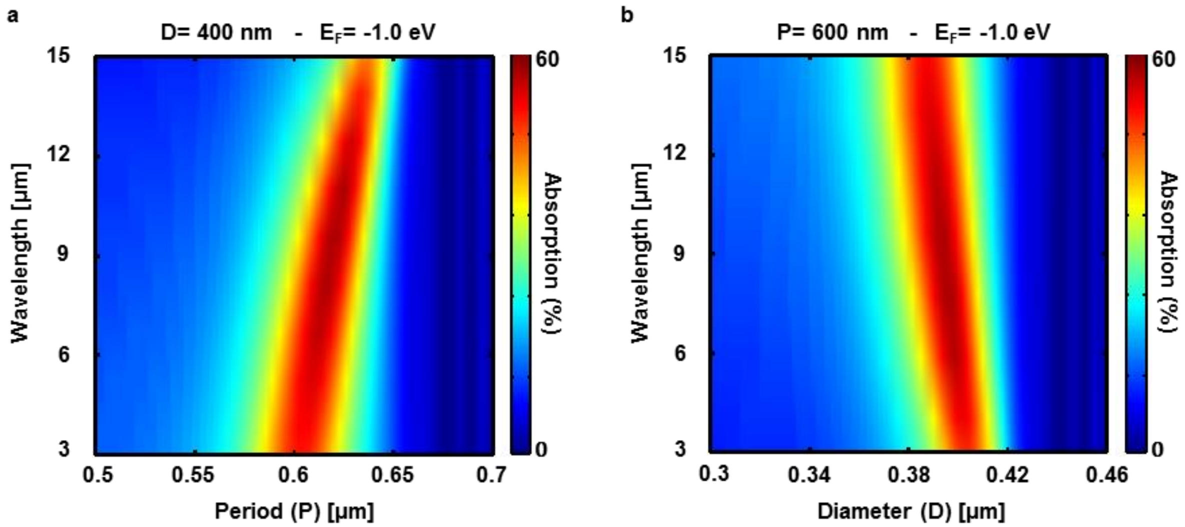

Supplementary Figure 6| The light absorption as a function of wavelength and period (a) diameter (b) for E_F= -1.0 eV.

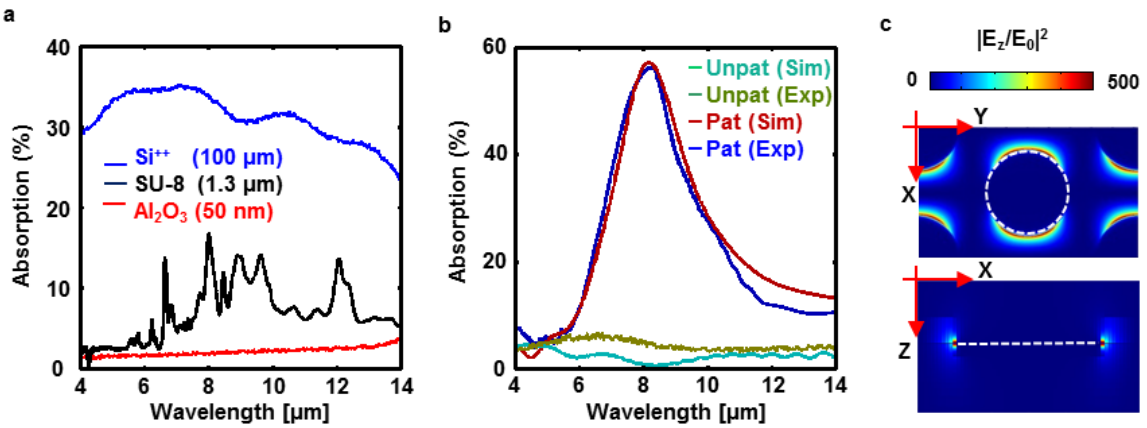

Supplementary Figure 7| (a) The measured light absorption of Si++ (100 µm), SU-8 (1.3 µm), and Al_2O_3 (50 nm) layers. (b) The optical absorption of the cavity-coupled unpatterned and patterned graphene with *P*= 600 nm, *D*= 400 nm, *L*= 1300 nm and E_F= -1 eV. (c) Top view and cross-section field profile at LSPR shows that the field intensity is maximum at graphene. [Pat – Patterned, Unpat – Unpatterned, Sim – Simulation and Exp – Experiment]

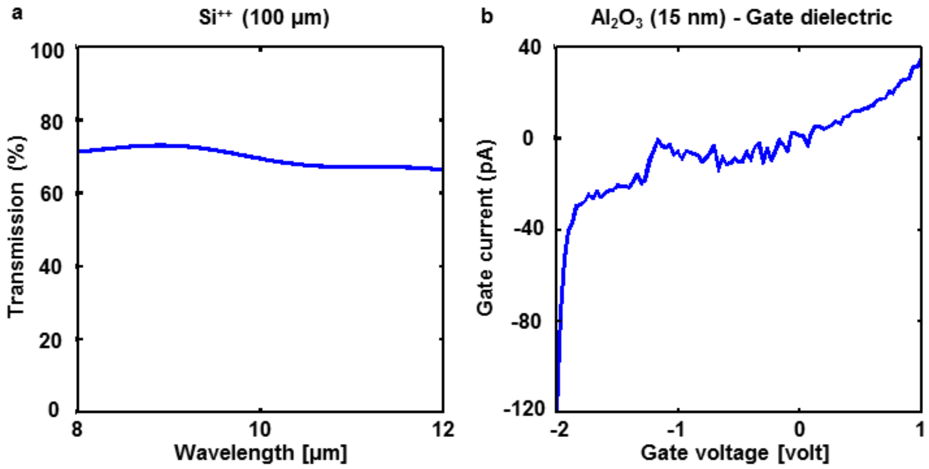

Supplementary Figure 8| Characterization of the Silicon and gate dielectrics. (a) The light transmission spectrum of Si^{++} with thickness of 100 µm. (b) The gate leakage electric current of the 15 nm thick layer of Al_2O_3.

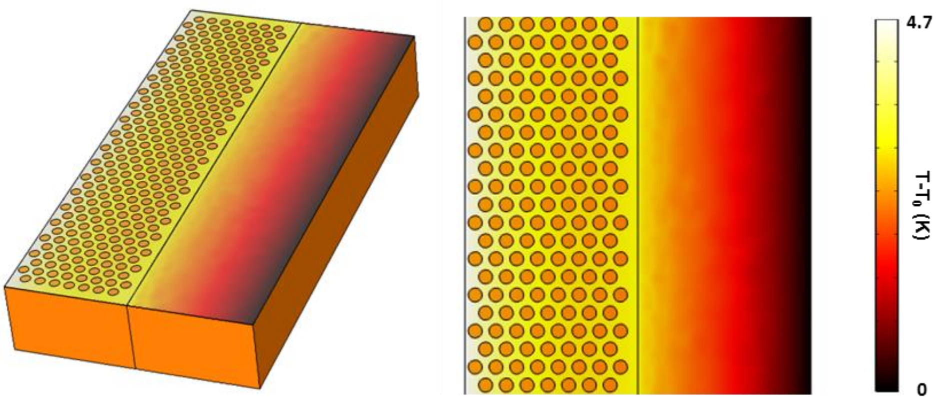

Supplementary Figure 9| The simulated temperature profile of the half-patterned graphene detector at E_F= -0.85 eV, λ_{res}= 8.15 μm and V_{SD}= 0.9 V.

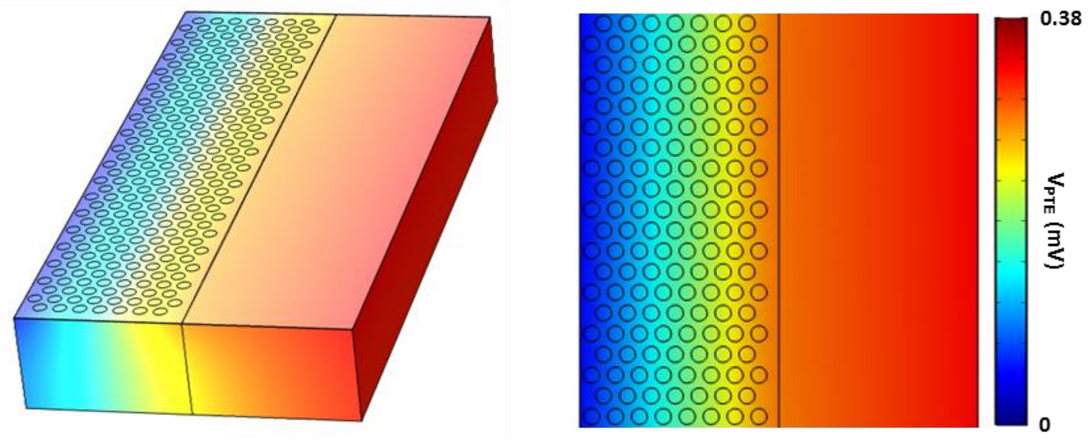

Supplementary Figure 10| The simulated potential profile of the half-patterned graphene detector at E_F= -0.85 eV, λ_{res}= 8.15 μm and V_{SD}= 0.9 V.

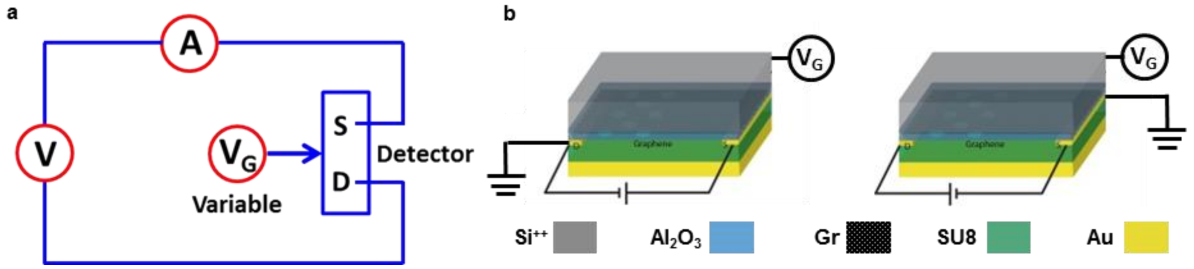

Supplementary Figure 11| Circuit diagram of the D.C measurement. (a) The circuit used to measure D.C response. (b) The schematic of the circuits used to measure the D.C currents correspond to the bias voltages with opposite polarities.

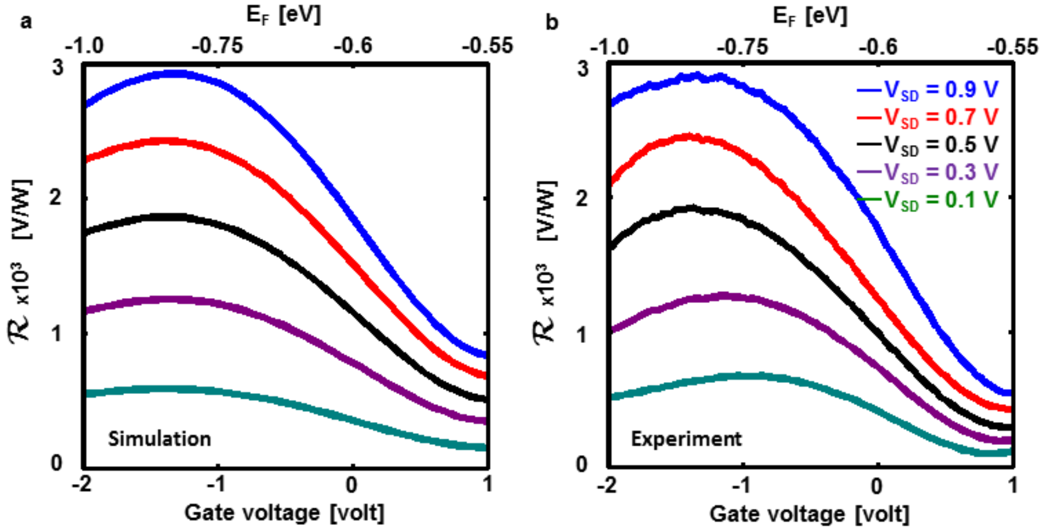

Supplementary Figure 12| The simulated (a) and measured (b) D.C responsivities of the half-patterned graphene sample as a function of gate-voltage for different bias voltages at P_{inc}= 153 nW and T_S= 295 K (a).

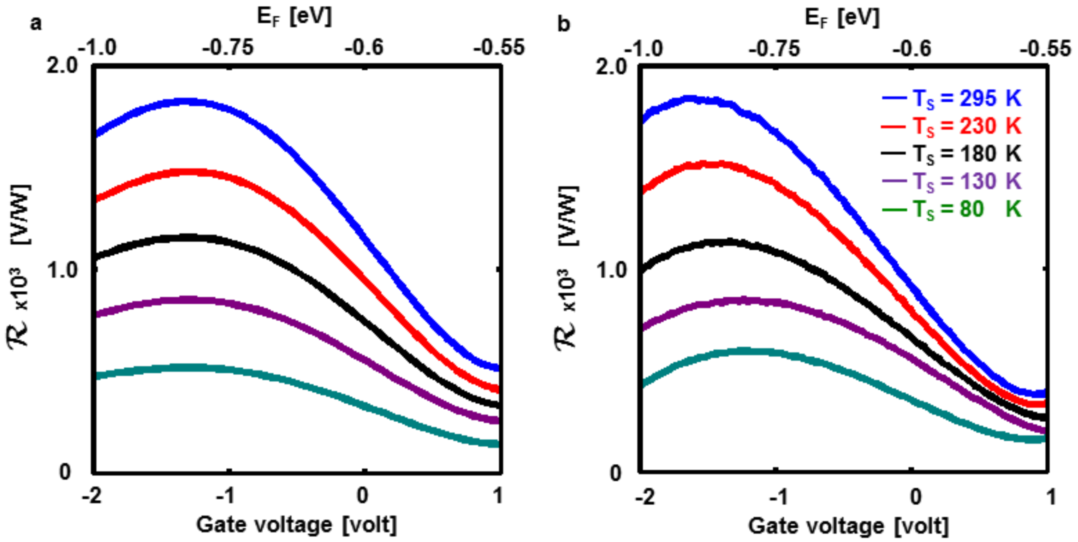

Supplementary Figure 13| The simulated (a) and measured (b) D.C responsivities of the half-patterned graphene sample as a function of gate-voltage for different substrate temperatures at P_{inc}= 153 nW and V_{SD}= 0.5 V.

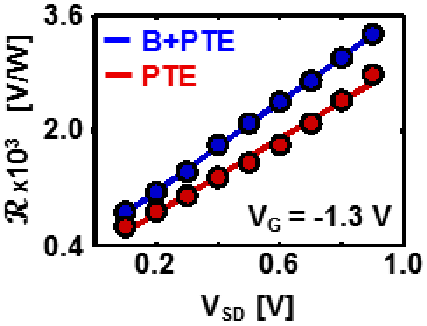

Supplementary Figure 14| D.C responsivity as a function of bias voltage for gate voltage, V_G = -1.3 V.

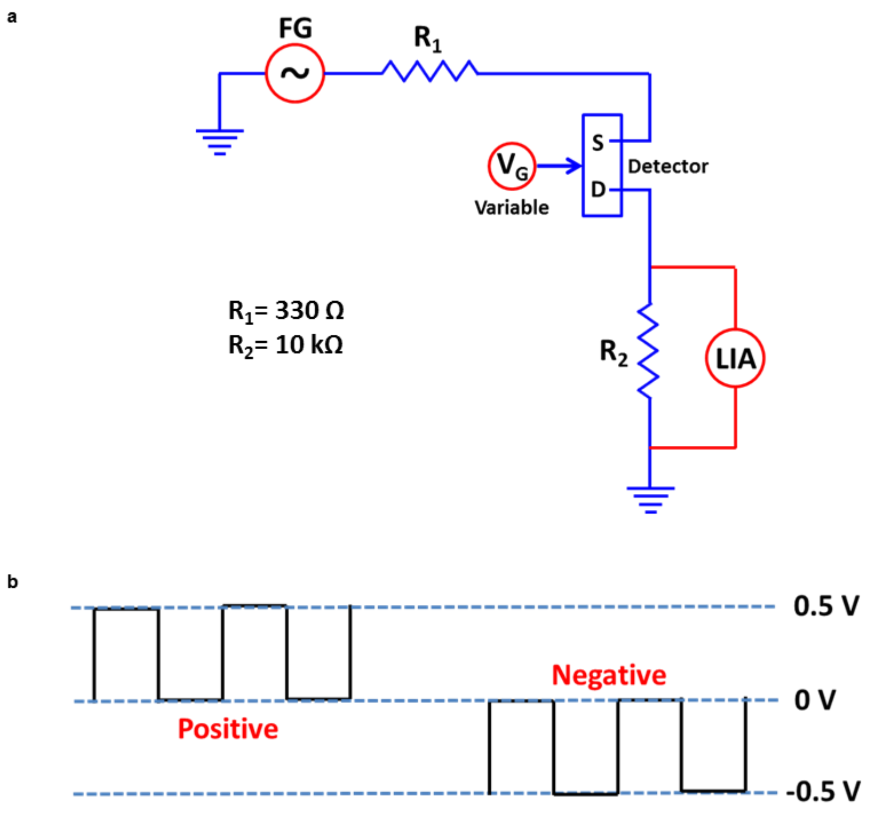

Supplementary Figure 15| Circuit diagram of the A.C measurement. (a) The circuit used to measure A.C response. (b) The positive and negative input biases. FG: Function generator; LIA: Lock-in-amplifier; V_G: Gate voltage

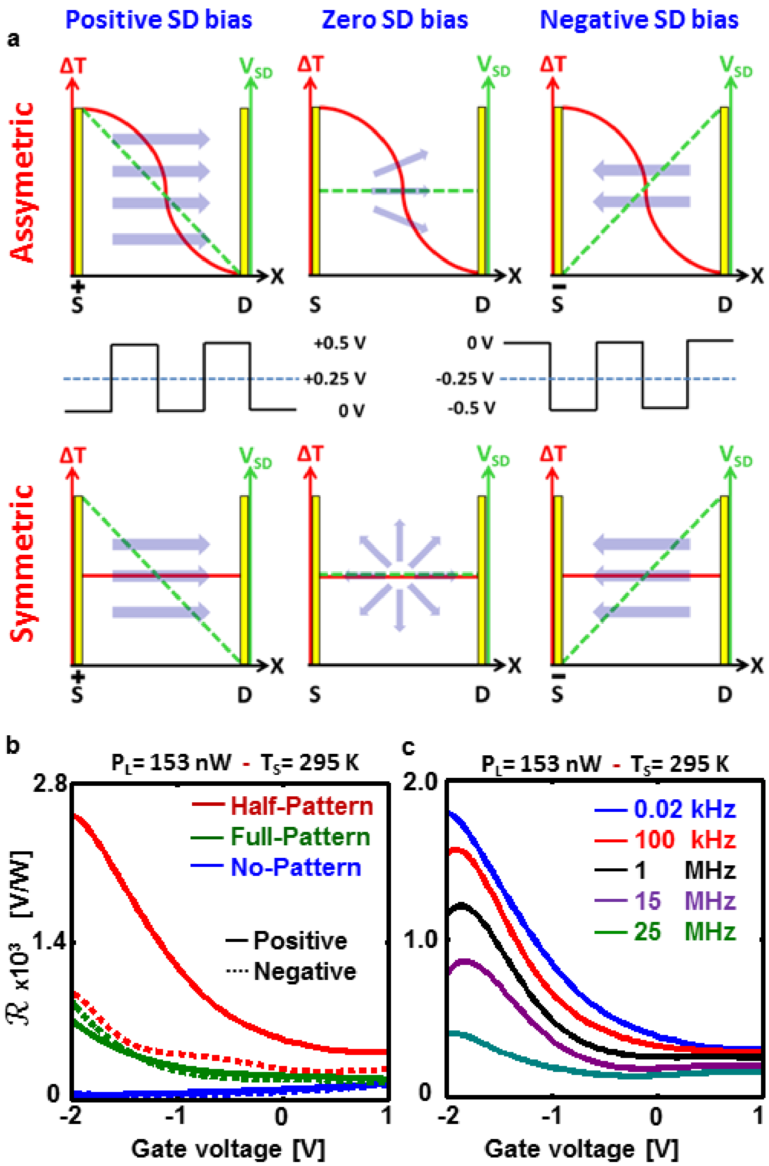

Supplementary Figure 16| A.C photoresponse. (a) Schematic showing the A.C photocurrent for different measurement conditions and detector devices. (b) A.C responsivity of the half-patterned (red), full-patterned (green) and unpatterned (blue) graphene detectors for the positive (solid) and negative (dashed) offsets (0.25 V) at f = 20 Hz. (c) A.C responsivity as a function of frequency for V_G= -2 V.

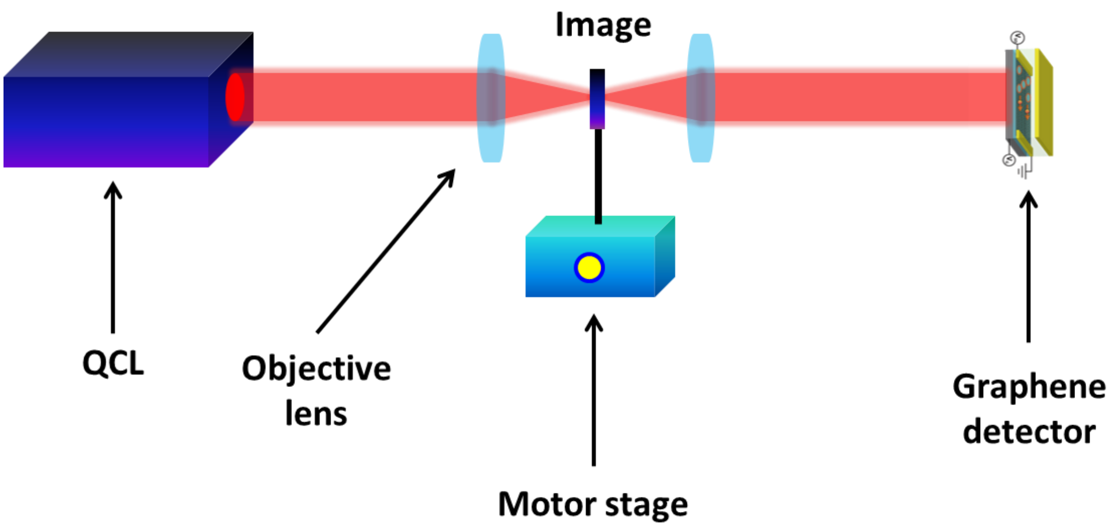

Supplementary Figure 17| Schematic of the single-pixel imaging setup.

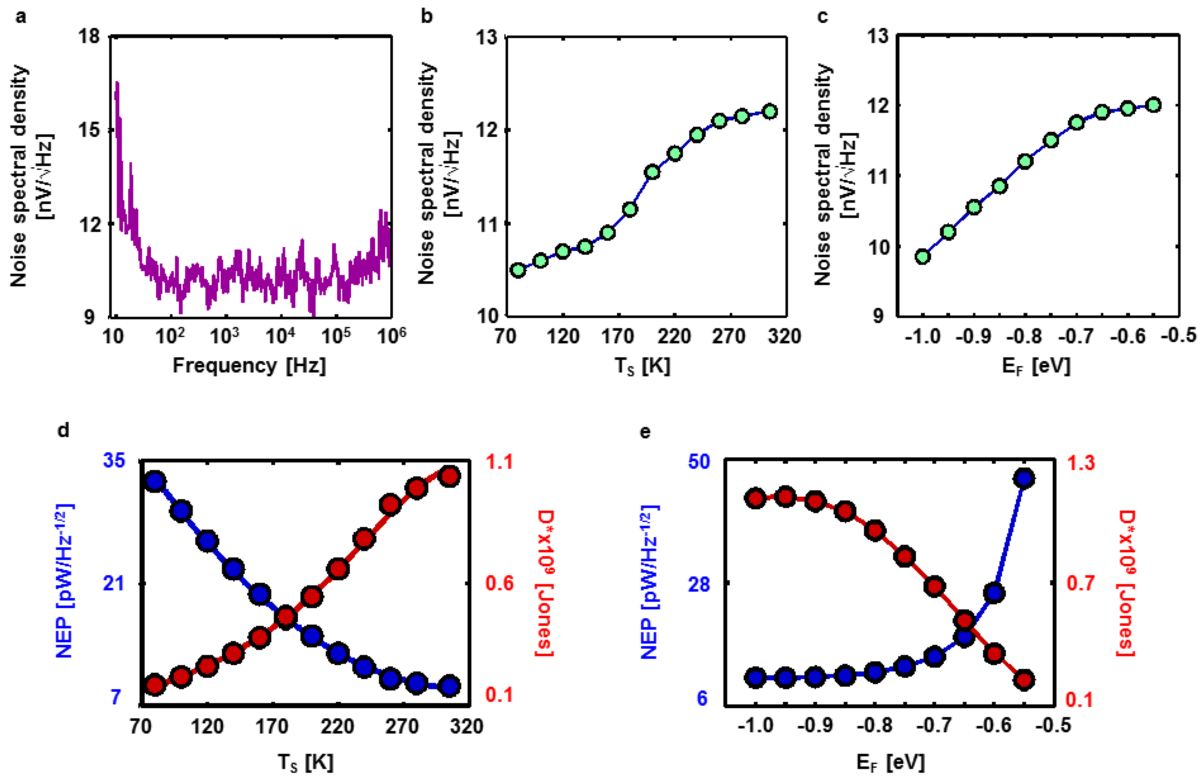

Supplementary Figure 18| (a) The noise spectral density of the graphene channel at room temperature and E_F= -1.0 eV. (b) The average noise spectral density as a function of temperature at E_F= -1.0 eV, and (c) as a function of Fermi energy at room temperature. (d) The noise equivalent power (NEP) and specific detectivity (D^*) of the graphene detector as a function of temperature at E_F= -1.0 eV, and (e) as a function of Fermi energy at room temperature.

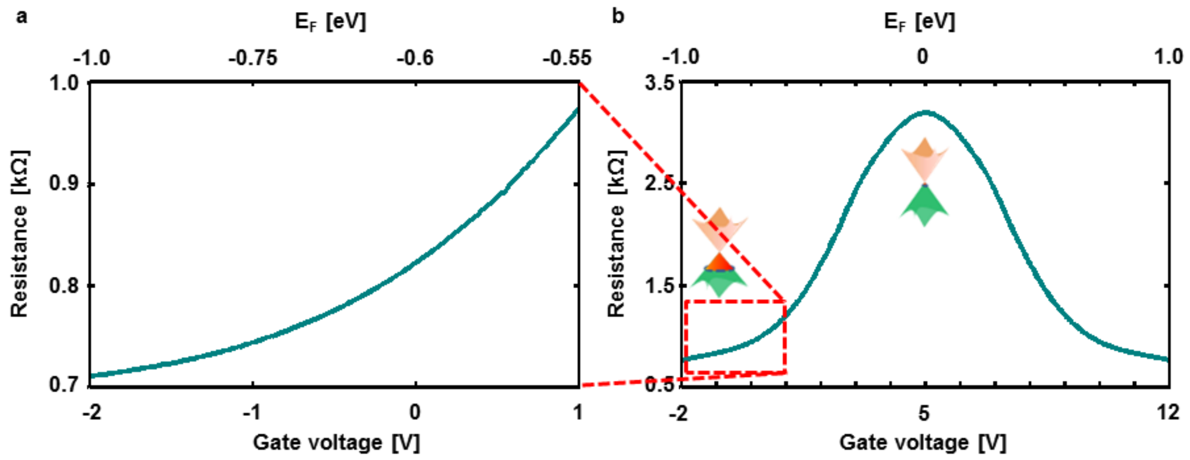

Supplementary Figure 19| The theoretical electrical resistance of monolayer graphene as a function of gate voltage for the self-doped graphene (a) and undoped graphene (b).

Supplementary References

1 Ferrari, A. C., Meyer, J. C., Scardaci, V., Casiraghi, C., Lazzeri, M., Mauri, F., Piscanec, S., Jiang, D., Novoselov, K. S., Roth, S. & Geim, A. K. Raman spectrum of graphene and graphene layers. *Phys. Rev. Lett.* **97**, 187401 (2006).

2 Safaei, A., Chandra, S., Leuenberger, M. N. & Chanda, D. Wide Angle Dynamically Tunable Enhanced Infrared Absorption on Large-Area Nanopatterned Graphene. *ACS Nano* **13**, 421-428 (2019).

3 Safaei, A., Chandra, S., Leuenberger, M. N. & Chanda, D. Tunable Enhanced Mid-Infrared Light Absorption in Graphene. *CLEO: Applications and Technology.* ATh3B. 3 (2017).

4 Safaei, A., Chandra, S., Leuenberger, M. N. & Chanda, D. Wide Angle Electronically Tunable Enhanced Light Absorption in Nanopatterned Graphene. *Bulletin of the American Physical Society.* F14.00008 (2019).

5 Song, J. C., Rudner, M. S., Marcus, C. M. & Levitov, L. S. Hot carrier transport and photocurrent response in graphene. *Nano Lett.* **11**, 4688-4692 (2011).

6 Cai, X., Sushkov, A. B., Suess, R. J., Jadidi, M. M., Jenkins, G. S., Nyakiti, L. O., Myers-Ward, R. L., Li, S., Yan, J., Gaskill, D. K., Murphy, T. E., Drew, H. D. & Fuhrer, M. S. Sensitive room-temperature terahertz detection via the photothermoelectric effect in graphene. *Nat. Nanotechnol.* **9**, 814-819 (2014).

7 Dollfus, P., Hung Nguyen, V. & Saint-Martin, J. Thermoelectric effects in graphene nanostructures. *Journal of physics. J. Phys. Condens. Matter* **27**, 133204 (2015).

8 Duan, J., Wang, X., Lai, X., Li, G., Watanabe, K., Taniguchi, T., Zebarjadi, M. & Andrei, E. Y. High thermoelectricpower factor in graphene/hBN devices. *Proc. Natl. Acad. Sci. U.S.A.* **113**, 14272-14276 (2016).

9 Hwang, E. H., Rossi, E. & Das Sarma, S. Theory of thermopower in two-dimensional graphene. *Phys. Rev. B* **80**, 235415 (2009).

10 Shautsova, V., Sidiropoulos, T., Xiao, X., Gusken, N. A., Black, N. C. G., Gilbertson, A. M., Giannini, V., Maier, S. A., Cohen, L. F. & Oulton, R. F. Plasmon induced thermoelectric effect in graphene. *Nat. Commun.* **9**, 5190 (2018).

11 Vazquez-Guardado, A., Safaei, A., Modak, S., Franklin, D. & Chanda, D. Hybrid coupling mechanism in a system supporting high order diffraction, plasmonic, and cavity resonances. *Phys. Rev. Lett.* **113**, 263902 (2014).

12 Chandra, S., Franklin, D., Cozart, J., Safaei, A. & Chanda, D. Adaptive Multispectral Infrared Camouflage. *ACS Photonics* **5**, 4513-4519 (2018).

13 Chanda, D., Safaei, A. & N. Leuenberger, M. Optical detector device with patterned graphene layer and related methods. *US Patent* App. 15/782,948 (2018).

14 Chanda, D., Modak, S., Lee, J., & Safaei, A. Optical Frequency-Selective Absorber-based Infrared Detector, Methods, and Applications. *US Patent* App. 15/538,746 (2018).

15 Safaei, A., Vázquez-Guardado, A., Franklin, D., Leuenberger, M. N. & Chanda, D. High-Efficiency Broadband Mid-Infrared Flat Lens. *Adv. Opt. Mater.* **6**, 1800216 (2018).

16 Safaei, A., Chandra, S., Vázquez-Guardado, A., Calderon, J., Franklin, D., Tetard, L., Zhai, L., Leuenberger, M. N. & Chanda, D. Dynamically tunable extraordinary light absorption in monolayer graphene. *Phys. Rev. B* **96**, 165431 (2017).

17 Murali, R., Yang, Y., Brenner, K., Beck, T. & Meindl, J. D. Breakdown current density of graphene nanoribbons. *Appl. Phys. Lett.* **94**, 243114 (2009).

18 Edgar, M. P., Gibson, G. M. & Padgett, M. J. Principles and prospects for single-pixel imaging. *Nat. Photonics* **13**, 13-20 (2018).
19 Fang, Y., Armin, A., Meredith, P. & Huang, J. Accurate characterization of next-generation thin-film photodetectors. *Nat. Photonics* **13**, 1-4 (2018).
