# Supplementary material for: Dirac plasmon-assisted asymmetric hot carrier generation for room-temperature infrared detection
Source: Nat Commun. 2019 Aug 2;10:3498. doi: 10.1038/s41467-019-11458-5 (PMC6677812; doi:10.1038/s41467-019-11458-5)
Supplement: Supplementary file 2 — Description of Additional Supplementary Files [file 41467_2019_11458_MOESM2_ESM.pdf]

Description of Additional Supplementary Files

File Name: Supplementary Movie 1

Description: The imaging result of UCF logo by using a single-pixel imaging method for different gate voltages.

File Name: Supplementary Movie 2

Description: The imaging result of Pegasus by using a single-pixel imaging method for different gate voltages.
